# Reply to: Diffusion anomaly in nanopores as a rich field for theorists and a challenge for experimentalists

**DOI:** 10.1038/s41467-024-49822-9

**Published:** 2024-07-08

**Authors:** Mingbin Gao, Jiamin Yuan, Zhiqiang Liu, Mao Ye, Anmin Zheng

**Affiliations:** 1 National Engineering Research Center of Lower-Carbon Catalysis Technology, Dalian National Laboratory for Clean Energy, Dalian Institute of Chemical Physics, Chinese Academy of Sciences, Dalian, People’s Republic of China; 2 State Key Laboratory of Magnetic Resonance and Atomic and Molecular Physics, National Center for Magnetic Resonance in Wuhan, Wuhan Institute of Physics and Mathematics, Innovation Academy for Precision Measurement Science and Technology, Chinese Academy of Sciences, Wuhan, People’s Republic of China

**Keywords:** Heterogeneous catalysis, Chemical physics

**replying to** S. Brandani et al. *Nature Communications* 10.1038/s41467-024-49821-w (2024)

Here we provide a comprehensive response to the Matter Arising by Brandani et al.^1^. They pointed out that the experimental data presented in Fig. 5e of our published article^2^ can be well-fitted by a first-order kinetic model (e.g., surface barriers^3^). Practically, the dual resistance model (DRM) can be used to fit the uptake process, reflecting the contributions of intracrystalline diffusion resistance and surface barriers with explicit physics meaning. In this reply, we illustrate the limitation of the dual-resistance model to decouple the surface barriers and intracrystalline diffusion based on the theorical analysis.

## Introduction

Many scientists have previously proposed some interesting diffusion mechanisms such as “floating molecule”^4^, “levitation effect”^5^, and so on. However, little work has been done to consider the influence of molecular degrees of freedom on diffusion in confined channels, especially for long-chain molecules. In our work, we proposed a scheme to control the pore size of zeolite channels to adjust the degree of freedom of molecules to achieve ultrafast diffusion, in which we adopted both the molecular dynamics (MD) simulations (additional computational details in Supplementary Note 2 of Supplementary Information) and diffusion experiments as the research methods.

## Results and discussion

**Fig. 1:**
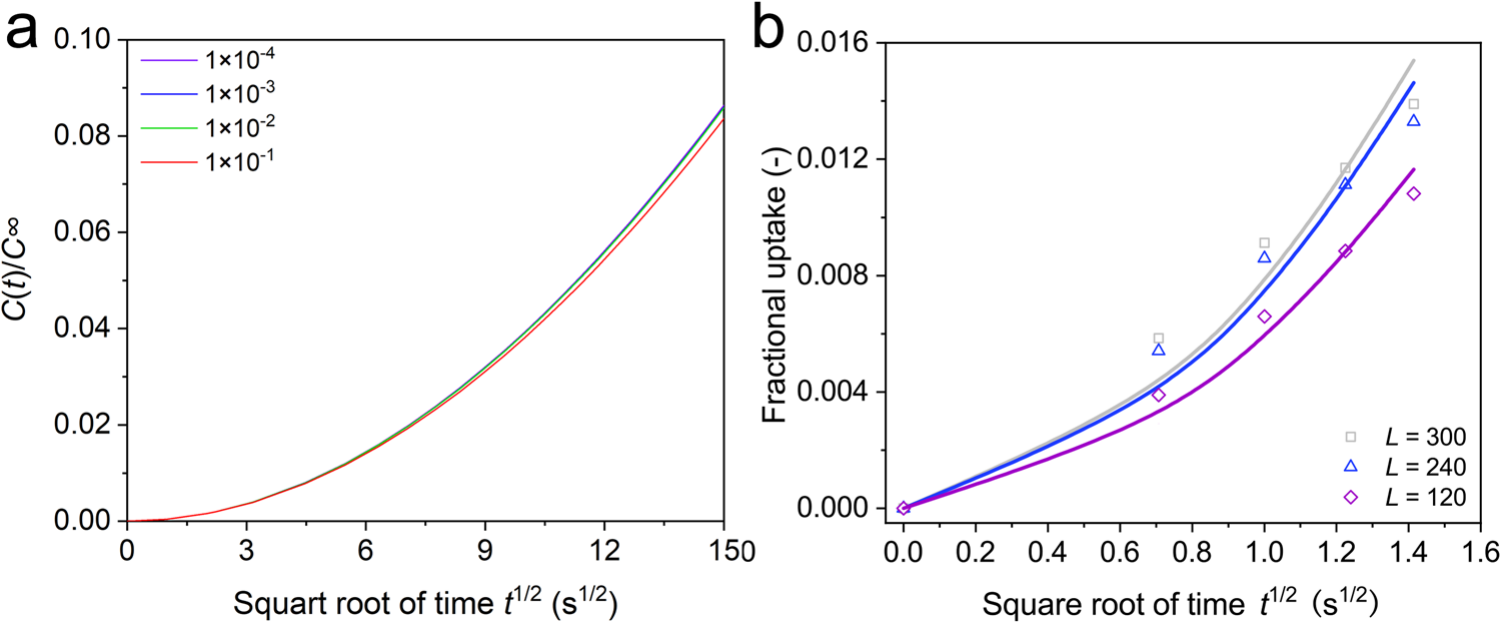
Analysis based on dual-resistance model to determine the application range. **a** Initial uptake rates by dual-resistance model for *L* = 1 × 10^−4^, 1 × 10^−3^, 1 × 10^−2^, and 1 × 10^−1^. **b** Initial uptake rates by dual-resistance model for *L* = 300, 240, and 120. The solid line is fitted by the equation (S1), and the discrete point is obtained by the equation (S2). Source data are provided as a Source Data file.

In our original work^2^, we measured uptake curves of *n*-C_12_ and *n*-C_4_ over different zeolites and used DRM to obtain the surface permeability and intracrystalline diffusivity. In this reply, we would like to illustrate the limitation of DRM. In Fig. 1a, b, we plotted the theoretical uptake curves with different values of *L* (*L* = *αl*/*D*, where *α* is the surface permeability, *D* is the intracrystalline diffusivity and *l* is the half-length of the crystal). In Fig. 1a, the value of *L* is regulated by the different value of *D* while keeping the same value of *α*. In Fig. 1a, the uptake rate decreases as the increase in *L* value (*L* = *αl*/*D*), which is resulted from the decrease in intracrystalline diffusivity. In Fig. 1b, the uptake rate increases as the increase in *L* value, which is resulted from the increase in surface permeability. In Fig. 1a, for the range of *L* from 10^−4^ to 10^−2^, the uptake curves are unaffected by the intracrystalline diffusivity. Therefore, it can be concluded that when the value of *L* is below 10^−1^, the significant dominance of surface barriers on mass transfer makes it difficult to decouple the intracrystalline diffusivity from uptake curves by use of the dual-resistance model. In Fig. 1b, the value of *L* is regulated by the different value of *α* while keeping the same value of *D*. We show when the value of *L* is larger than 200 (for the sample interval ~1 s), it is difficult to determine the surface permeability by fitting the initial uptake curves.

**Fig. 2:**
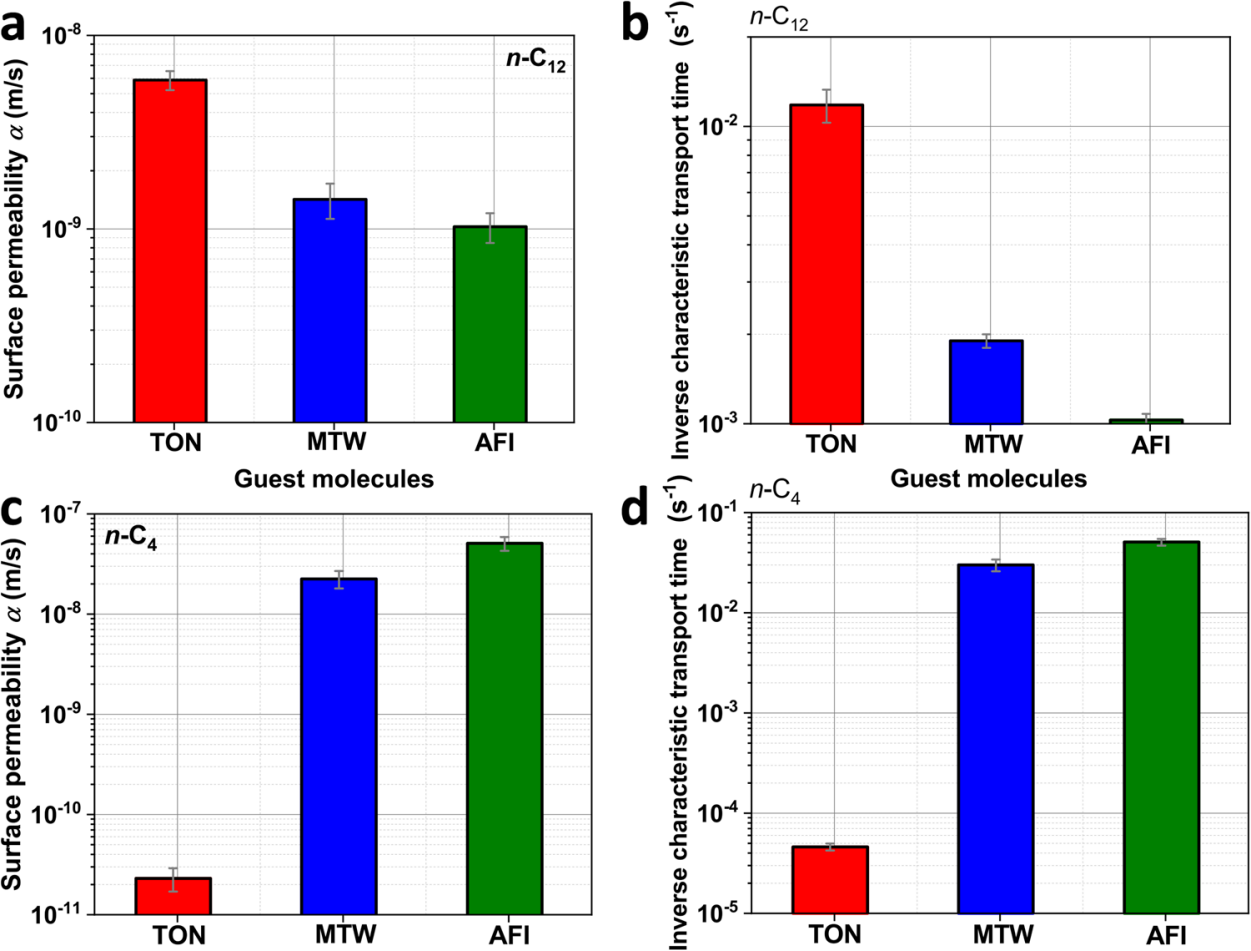
Uptake results of n-C12 and n-C4 in zeolites with small crystal size. **a** The surface permeability and **b** inverse characteristic mass transport time (fitting by the first-order exponential model) of *n*-C_12_ over TON-S, MTW-S, and AFI-S samples at 298 K^2^. **c** The surface permeability and **d**, inverse characteristic diffusion mass transport time (fitting by the first-order exponential model) of *n*-C_4_ over TON-S, MTW-S, and AFI-S samples at 298 K^2^. The infrared microscopy (IRM) experimental conditions are as follows: the flowrate is 35 ml/min and the loading of zeolites in cell is ~1 mg. The intelligent gravimetric analyzer (IGA) experimental conditions are as follows: the loading of zeolites in IGA sample cell is 12 mg and the pressure of *n*-C_4_ changes from 0 to 4 mbar. Figure 2 were refitted from ref. ^2^. The value of bar graphs in (**a**, **c**) is obtained by fitting experimental data in ref. ^2^ by equation (S1). The value of bar graphs in (**b**, **d**) is obtained by fitting experimental data in ref. ^2^ by equation (S3). The error band is the standard error of fitting by equation (S1) and (S3). Source data are provided as a Source Data file.

**Fig. 3:**
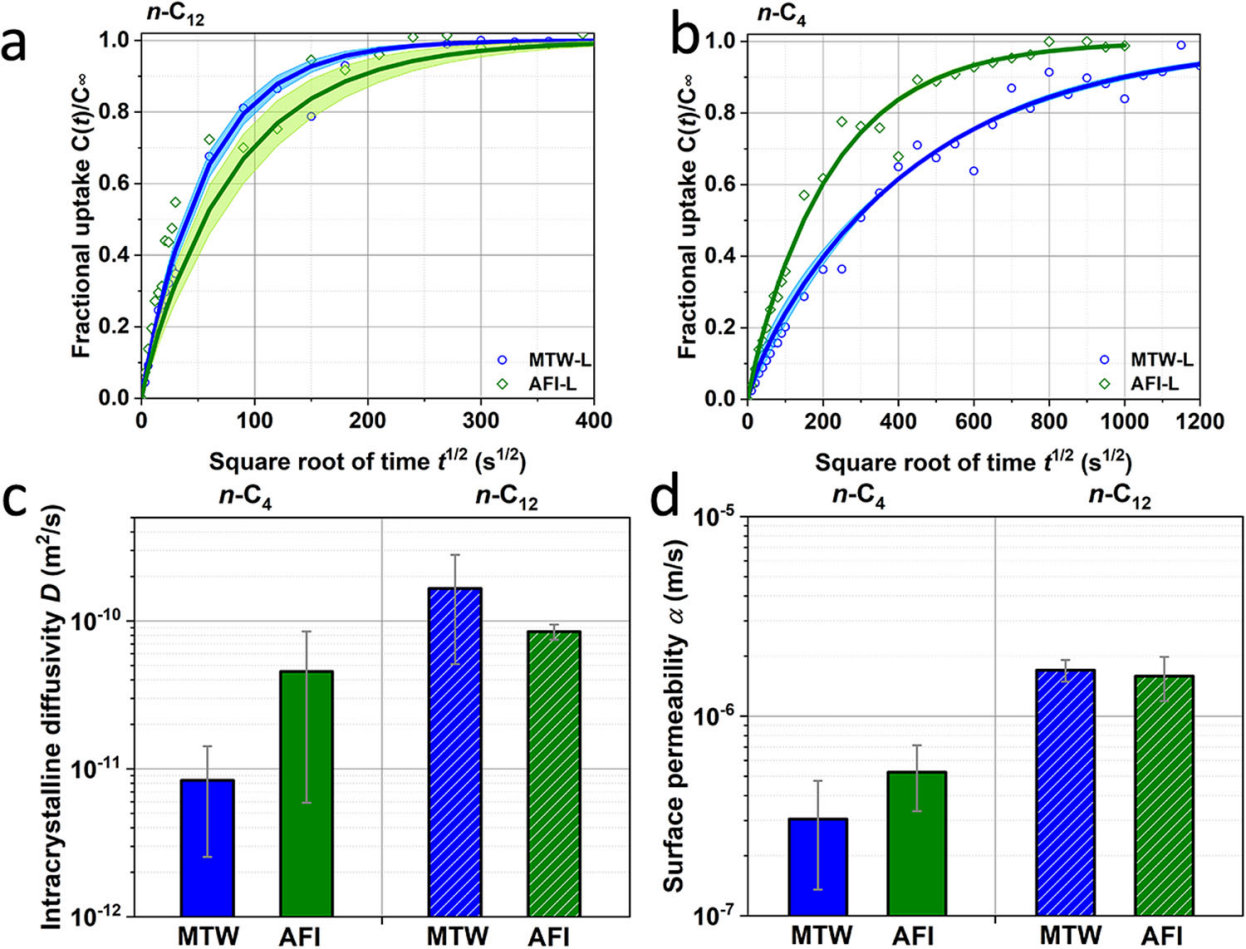
Uptake results of n-C12 and n-C4 in zeolites with large crystal size. The uptake curves and fitting results of **a** *n*-C_12_ and **b** *n*-C_4_ over AFI-L and MTW-L samples. The infrared microscopy (IRM) experimental conditions are as follows: the flowrate is 35 ml/min and the loading of zeolites in cell is ~1 mg. **c** The intracrystalline diffusivity of *n*-C_12_ (*L*_*n*-C12,MTW_ = 0.77 ± 0.46 and *L*_*n*-C12,AFI_ = 1.45 ± 0.50) and *n*-C_4_ (*L*_*n*-C4,MTW_ = 3.44 ± 2.56 and *L*_*n*-C4,AFI_ = 1.61 ± 1.22) in AFI-L and MTW-L samples. **d** The surface permeability of *n*-C_12_ and *n*-C_4_ in AFI- L and MTW-L samples. **a**, **b** The solid line is fitted by the equation (S2), and discrete point is measured by the experiments. The error band is the standard error of experimental results of IGA measurements. Source data are provided as a Source Data file.

In our published paper^2^, we measured the diffusivities of *n*-C_12_ and *n*-C_4_ over TON, MTW, and AFI zeolites with small crystal sizes (≤ 2 μm). The results indicate that, in addition to intracrystalline diffusion, the presence of surface barriers can be observed. Therefore, we employed DRM to fit uptake curves, as DRM can be employed to decouple the intracrystalline diffusivity and surface permeability for a variety of guest molecules in different zeolites^6–9^. Remi et al.^6^ found the mass transport of methanol and butanol over individual H-SAPO-34 can be surface barriers-controlled based on the interference microscopy results in which they decoupled the intracrystalline diffusivity and surface permeability by DRM. Discrepant infrared signals in *n*-C_12_ presented in Supplementary Fig. 14 of our published paper^2^ show the different confinement effect imposed by intracrystalline-frameworks of TON, MTW and AFI zeolites. Therefore, we employed DRM to fit the intracrystalline diffusivity *D* and surface permeability *α*. Brandani et al. found it is not appropriate to use the value of intracrystalline diffusivity to present the experimental results due to the strong effect of surface barriers due to the small value of *L*. In this reply, based on our published data, we further presented the surface permeability in Fig. 2a, c. In Fig. 2b, We also calculated the inverse characteristic mass transport time (fitting by the first-order exponential model^6^ in Eq. 3) of *n*-C_12_ over TON-S, MTW-S and AFI-S samples is 1.18 × 10^−2^, 1.90 × 10^−3^, and 1.03 × 10^−3 ^ s^−1^, respectively. In Fig. 2d, the inverse characteristic mass transport time of *n*-C_4_ over TON-S, MTW-S and AFI-S samples is 4.60 × 10^−5^, 3.00 × 10^−2^ and 5.07 × 10^−2 ^ s^−1^, respectively. These results can obtain the same trend as found in MD simulations. In order to ensure the reliable fitting by DRM, zeolites with large MTW and AFI crystals (>100 μm) are adopted for experiments. In Fig. 3a, b, the uptake data over zeolites with large crystal sizes can be well-fitted by DRM, which shows the applicability of DRM for  the L range between 0.1-100. The effect of signal noise on the fitting errors, intracrystalline diffusivity and surface permeability has been examined. We found that the signal noise has significant effect on the fitting results (*L* value, intracrystalline diffusivity and surface permeability). In Fig. 3c, d, the average value and standard deviation of intracrystalline diffusivity and surface permeability are shown. We noticed that the uptake curves measured by microimaging techniques usually accompany with signal noise^6,10^. Therefore, how to determine the representative results under the signal noise effect is well-worth as an independent work. The average value of intracrystalline diffusivity and surface permeability can validate the trends obtained from MD simulations.

In the practical diffusion measurements over zeolite materials, the measured effective diffusivity can be affected by many factors besides zeolite frameworks, e.g., surface defects^3^, internal interfaces, and intergrowth structures^11,12^. At present, researchers usually compare the trends of experimental results and MD simulations to obtain a systematic understanding at both macro and micro levels^13^. So far, quantitative comparison between MD simulations and experimental diffusion measurements is a non-trivial task and remains a big challenge.

## Methods

See Supplementary Information for details.

## Supplementary information

Supplementary Information

## Source data

source data

## Data Availability

Source data are provided with this paper.
