# Publisher Correction: Reply to: Diffusion anomaly in nanopores as a rich field for theorists and a challenge for experimentalists

**DOI:** 10.1038/s41467-024-52103-0

**Published:** 2024-09-02

**Authors:** Mingbin Gao, Jiamin Yuan, Zhiqiang Liu, Mao Ye, Anmin Zheng

**Affiliations:** 1 National Engineering Research Center of Lower-Carbon Catalysis Technology, Dalian National Laboratory for Clean Energy, Dalian Institute of Chemical Physics, Chinese Academy of Sciences, Dalian, People’s Republic of China; 2 State Key Laboratory of Magnetic Resonance and Atomic and Molecular Physics, National Center for Magnetic Resonance in Wuhan, Wuhan Institute of Physics and Mathematics, Innovation Academy for Precision Measurement Science and Technology, Chinese Academy of Sciences, Wuhan, People’s Republic of China

**Keywords:** Heterogeneous catalysis, Chemical physics

Correction to: *Nature Communications* 10.1038/s41467-024-49822-9, published online 08 July 2024

A Peer Review File associated with this Article was originally published online, but was removed shortly after publication, as *Nature Communications* does not operate transparent peer review for Matters Arising articles. The original article has been corrected.

