# Supplementary material for: Reply to: Diffusion anomaly in nanopores as a rich field for theorists and a challenge for experimentalists
Source: Nat Commun. 2024 Jul 8;15:5722. doi: 10.1038/s41467-024-49822-9 (PMC11231131; doi:10.1038/s41467-024-49822-9)
Supplement: Supplementary file 1 — Supplementary Information [file 41467_2024_49822_MOESM1_ESM.pdf]

Supplementary Information to "Reply to: Diffusion anomaly in nanopores as a rich field for theorists and a challenge for experimentalists"

Gao et al.

Supplementary Methods

Supplementary Note 1. Experimental details

In our published paper[1], we employed commercial TON (TON-S, 1 μm, H-ZSM-22), MTW (MTW-S, 1.5 μm, H-ZSM-12) and AFI (AFI-S, 2 μm, H-SAPO-5) zeolites to measure uptake rates[2] of n-C_{12} and n-C_4 (Supplementary Figure 1). We employed DRM to fit the intracrystalline diffusivity D and surface permeability α. As shown in Figure 2a and c, we first obtained α (Figure 2a for n-C_{12} and Figure 2c for n-C_4) by fitting the initial uptake curve using equation[3]

$$\frac{c_t}{c_\infty}|^{t\to 0} = \frac{\alpha}{l}\left(\sqrt{t}\right)^2, \qquad \text{equation (S1)}$$

where c_t/c_∞ is the relative uptake loading of guest molecules, t the uptake time and l the half-length of crystal. Based on the obtained surface permeability, we can obtain D by fitting the whole uptake curve by DRM

$$\frac{c_t}{c_\infty} = 1 - 2L^2 \sum_{n=1}^{\infty} \frac{e^{-\beta_n^2 Dt/l^2}}{[\beta_n^2 + L(L+1)]\beta_n^2}, \qquad \text{equation (S2)}$$

where $L = \alpha l/D = \beta_n \tan\beta_n$ is the ratio of characteristic time of intracrystalline diffusion to that of surface barriers.

The first-order exponential model

$$\frac{c_t}{c_\infty} = 1 - \exp\left(-\frac{t}{t_c}\right), \qquad \text{equation (S3)}$$

where t_c is the inverse characteristic mass transport times.

We synthesized MTW and AFI zeolites with large crystal size following the recipes in reference[4,5]. In the Supplementary Figure 2, the morphologies and crystal sizes of zeolites were observed by FE-SEM Hitachi SU8020. The uptake rates of n-C_{12} and n-C_4 over MTW (~ 120 μm) and AFI (~ 150 μm) zeolites were measured by IRM as shown in Figure 3a and b, respectively. The surface permeability and intracrystalline diffusivity were fitted by the similar procedure described above.

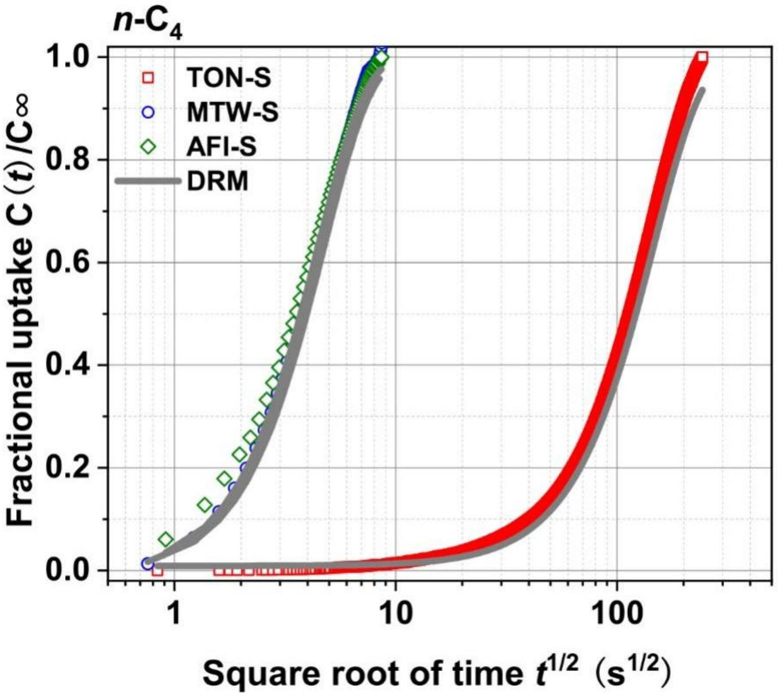

Supplementary Figure 1. The uptake curve and DRM fitting results of *n*-C_4 over TON-S, MTW-S and AFI-S samples at 298 K. The solid line is fitted by the equation (S2) and discrete point is measured by the experiments. Source data are provided as a Source Data file.

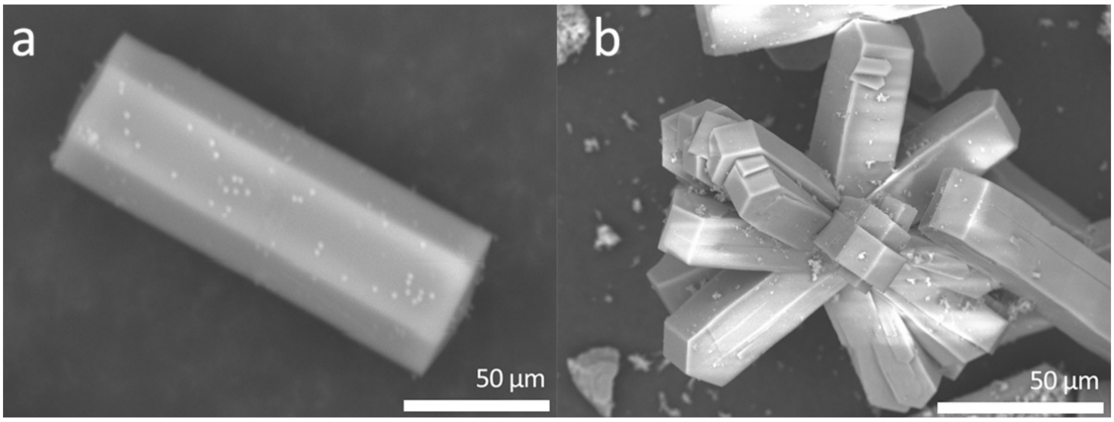

Supplementary Figure 2. The morphology of **a**, AFI-L and **b**, MTW-L samples measured by cold-field emission scanning electron microscopy.

Supplementary Note 2. Simulations details

To investigate the effect of pore-size on molecular diffusion, we built a series of sub-nanometer channel models of different pore-size. Because studies have shown

that for zeolite systems, the interaction between atom O and alkanes occupies the main part[6,7], therefore, we used the parameters of atom O to build model (as shown Supplementary Figure 3a). As we know, the mean square displacement (MSD) of adsorbates is defined via the following equation[8]:

$$\mathrm{MSD}(\tau) = \frac{1}{N_m}\sum_{i}^{N_m}\frac{1}{N_\tau}\sum_{t_0}^{N_\tau}[r_i(t_0+\tau) - r_i(t_0)]^2 \qquad \text{equation (S4)}$$

where N_m represents the number of gas molecules, N_τ is the number of time origins used in calculating the average, and r_i is the coordinate of the i-th molecule. According to the equation, it can be seen that the accuracy of MSD calculation is related to the number of molecules and MD simulation time, which can be improved by increasing the number of molecules or increasing the simulation time, and the latter is used in this work. The MSD with different simulation times is shown in the following Supplementary Figure 3b. Obviously, the longer the simulation time, the better the linearity of the MSD. As we mentioned in the methods section, each MD simulation was equilibrated at 2×10^6 steps, and then 4×10^7 production steps were recorded for the calculation of diffusion coefficients of adsorbate molecules. The timestep is 0.5 fs. The trajectories were recorded every 1000 steps. Furthermore, 3 independent MD simulations for each system were conducted for better statistics, the diffusion coefficients were calculated by fitting the linear region of MSD using a least-square fit (as shown in Supplementary Figure 3c). In addition, the slope of the MSD as a function of time determines the self-diffusion coefficient (D_s) defined according to the Einstein relationship, as shown below[8]:

$$\mathrm{MSD}(\tau) = 2nD_s\tau + b \qquad \text{equation (S5)}$$

In which, n is the dimension of frameworks (n = 1 for 1-D diffusion).

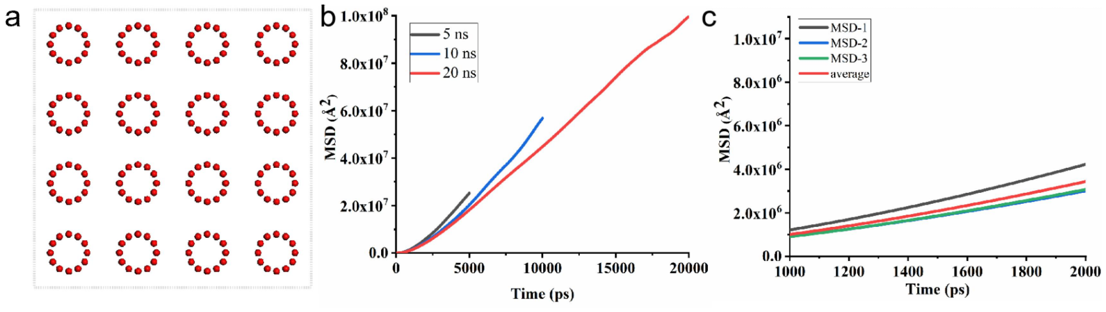

Supplementary Figure 3. a, The model of sub-nanometer channels composed of oxygen atoms, the MSD of $C_{12}H_{26}$ molecules inside 8 Å channel **b**, with different simulation time and **c.** the fitting linear region of 3 independent simulations within 20 ns. Source data are provided as a Source Data file.

Supplementary References

1 Yuan, J. *et al.* Hyperloop-like diffusion of long-chain molecules under confinement. *Nat. Commun.* **14**, 1735, doi:10.1038/s41467-023-37455-3 (2023).

2 Wang, J.-Y., Mangano, E., Brandani, S. & Ruthven, D. M. A review of common practices in gravimetric and volumetric adsorption kinetic experiments. *Adsorption* **27**, 295-318, doi:10.1007/s10450-020-00276-7 (2021).

3 Gao, M. *et al.* Direct quantification of surface barriers for mass transfer in nanoporous crystalline materials. *Commun. Chem.* **2**, 43-52, doi:10.1038/s42004-019-0144-1 (2019).

4 Liu, J. *et al.* Carbon dots in zeolites: A new class of thermally activated delayed fluorescence materials with ultralong lifetimes. *Sci. Adv.* **3**, e1603171, doi:10.1126/sciadv.1603171 (2017).

5 Ritsch, S. *et al.* High-Resolution Electron Microscopy Study of ZSM-12 (MTW). *Chem. Mater.* **10**, 3958-3965, doi:10.1021/cm980410a (1998).

6 Dubbeldam, D. *et al.* United Atom Force Field for Alkanes in Nanoporous Materials. *J. Phys. Chem. B* **108**, 12301-12313, doi:10.1021/jp0376727 (2004).

7 Dubbeldam, D. *et al.* Force Field Parametrization through Fitting on Inflection Points in Isotherms. *Phys. Rev. Lett.* **93**, 088302, doi:10.1103/PhysRevLett.93.088302 (2004).

8 Frenkel, D. & Smit, B. *Understanding molecular simulation: from algorithms to applications*. Vol. 1 (Elsevier, 2001).
